# Brace for Impact: A Retrospective Analysis of the Modified Broström-Gould Procedure With and Without Internal Brace Augmentation

**DOI:** 10.7759/cureus.44563

**Published:** 2023-09-02

**Authors:** David J Flaherty, Jamie McGuigan, Samuel E Cullen, Anand Pillai

**Affiliations:** 1 Trauma and Orthopaedics, Wythenshawe Hospital, Manchester, GBR

**Keywords:** modified broström-gould, full weight bearing, postoperative pain, chronic lateral ankle instability, atfl, internal brace

## Abstract

Background

Chronic lateral ankle instability (CLAI) is caused by lateral ankle ligament weakness or rupture secondary to recurrent sprains. The surgical management has traditionally involved a modified Broström-Gould (MBG) procedure with or without internal brace (IB) augmentation. This study aims to demonstrate the improved outcomes for patients undergoing an MBG procedure with IB augmentation for CLAI.

Methodology

A retrospective analysis was performed among 40 patients undergoing an MBG procedure with or without IB for CLAI at a large teaching hospital between January 2012 and June 2019. Functional outcomes were measured using the Manchester-Oxford Foot Questionnaire (MOxFQ). Clinic letters were reviewed to assess additional outcomes including postoperative complications, revision surgery rate, time in a plaster cast, and time to full weight-bearing.

Results

A total of 23 patients were included in the study, with seven undergoing both MBG and IB procedures and 16 undergoing MBG intervention alone. The average age was 37.1 years in the IB group and 35.7 years in the MBG group. The mean MOxFQ overall raw scores (10.9 vs. 33.6, p < 0.016), standing and walking MOxFQ subscale (4 vs. 15.2, p < 0.012), pain MOxFQ subscale (4.86 vs. 10.9, p < 0.042), and social interaction subscale (2 vs. 7.5 p < 0.023) all showed significantly better results for the IB group versus the MBG group. Patients in the IB group had significantly less number of weeks in plaster than the MBG group and were able to fully weight bear sooner (4.14 vs. 6, p < 0.01). The MBG group suffered a postoperative complication in seven patients compared to zero in the IB group (p < 0.057). There were three re-ruptures in the MBG group requiring further revision surgery compared to zero in the IB group (3 vs. 0, p < 0.53).

Conclusions

MBG surgery with IB augmentation for CLAI appears to have better outcomes in terms of overall function and may have fewer overall complications. The IB group displayed a lower recurrence of pain, less time in a plaster cast, and a quicker return to walking.

## Introduction

Chronic lateral ankle instability (CLAI) is a painful and debilitating condition caused by lateral ankle ligament weakness or rupture secondary to recurrent sprains or an acute injury [1]. The lateral ligament consists of the anterior talofibular ligament (ATFL), the posterior talofibular ligament (PTFL), the calcaneofibular ligament (CFL), the lateral talocalcaneal ligament (LTCL), and the syndesmotic complex [2]. Lateral ankle sprains are one of the most common musculoskeletal injuries occurring more frequently than medial ligament injuries due its weaker anatomical structure [3]. Lateral ligament injuries commonly occur due to an inversion mechanism of injury during sporting activity [1]. A high percentage (12-40%) of patients with a lateral ligament sprain will go on to develop CLAI [4].

Patients with CLAI report pain, recurrent swelling, and difficulty walking and standing for long periods, especially on uneven surfaces [5]. Most patients also report recurrent instability of the ankle, which can impact both social interactions and sporting activities [6]. A period of conservative management is first-line treatment including rest, anti-inflammatory medications, external bracing, and guided physiotherapy [5,7]. However, some patients with debilitating instability and pain may require surgical fixation which can improve functional outcomes and reduce secondary development of osteoarthritis [4,8,9].

Although many surgical techniques have been trialed for CLAI, the modified Broström-Gould (MBG) technique has been widely accepted as a beneficial biomechanical repair that improves strength and reduces re-rupture rates [10]. The MBG technique involves a suture repair of both the ATFL and CFL with extra reinforcement involving the LTCL and inferior extensor retinaculum [11].

However, recently, the technique has been criticized due to new evidence suggesting there are increased re-rupture rates in high ankle stress patients such as athletes or obese patients [7]. An internal brace (IB) technique was hypothesized to augment the MBG repair, and, subsequently, aid its stability and reduce re-rupture rates [10,12]. IB augmentation technique involves using a SwiveLock anchor pre-loaded with FiberTape inserted into the ATFL footprint on the distal fibula and the insertion point on the talus. This FiberTape SwiveLock anchor works to augment the traditional ATFL repair.

This retrospective case analysis aims to investigate local outcomes of MBG alone versus MBG with IB on both functional and mechanical outcomes and compare with previously published evidence [7,10,13]. Moreover, we aim to demonstrate for the first time in the literature, the time in plaster cast and time to full weight-bearing for both surgical techniques.

## Materials and methods

We performed a single-center retrospective analysis of all patients undergoing an MBG procedure with or without IB for CLAI at a large university teaching hospital (Wythenshawe Hospital) in Manchester, UK, between January 2012 and June 2019.

Study participants

All patients underwent either an MBG operation alone or an MBG procedure with IB augmentation. Data were collected from three consultant foot and ankle surgeons working within the department. Exclusion criteria included any revision procedures, any previous foot or ankle surgery, patients less than 18 years of age, and those who were either lost to follow-up or declined to complete the functional outcome questionnaire.

Data were collected from operation notes, clinic letters, and X-rays on the hospital’s electronic patient software as well as a functional outcome questionnaire which was completed retrospectively. Hospital approval was obtained for all aspects of this study in accordance with hospital policies.

Outcome measures

**Figure 1:**
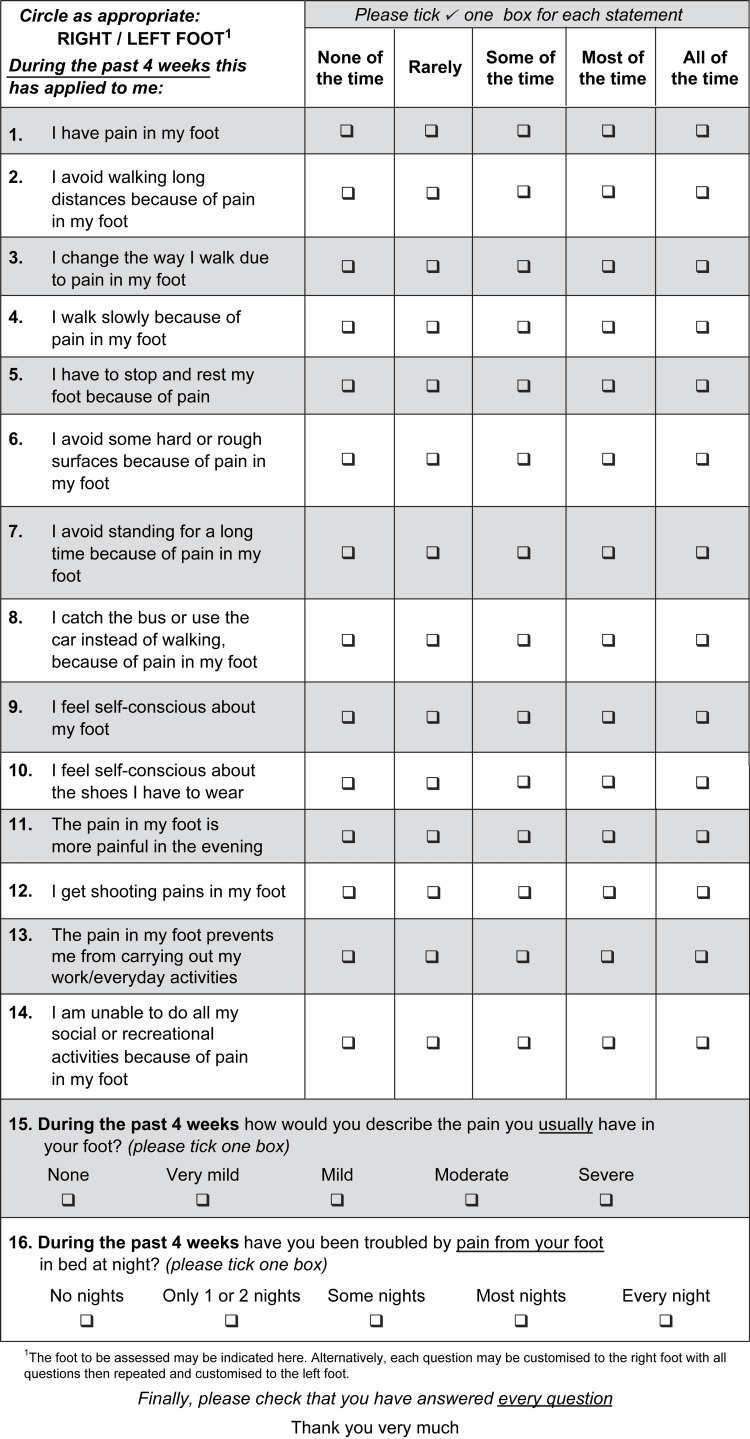
The Manchester-Oxford Foot Questionnaire (MOxFQ).

Data collected included functional outcomes which were measured using the Manchester-Oxford Foot Questionnaire (MOxFQ) (Figure 1). MOxFQ is a validated 16-point functional outcome measure that gives an overall index score as well as specific results for three subgroups of pain, standing/walking, and social interaction [14]. MOxFQ uses a five-point Likert scale for each question ranging from 0 (none of the time) to 5 (all of the time) [14]. The median time to completion of MOxFQ questionnaires and assessment of complications postoperatively was calculated for both MBG and IB groups.

Other outcome measures included patient demographics, time in a plaster cast, time to full weight-bearing, complication rates, time in an air cast boot, revision/re-rupture rates, and time spent in the hospital postoperatively.

Statistical analysis

Data were recorded and analyzed using Microsoft Excel (Microsoft Corp., Redmond, WA, USA). Continuous variables are presented as frequency (N), percentages (%), calculated means, and domain raw results (0-4) for the MOxFQ (0 = none of the time, 1 = rarely, etc.). Results were compared using the unpaired t-test. Categorical variables are presented as frequency (N) and percentages (%) and compared using Fisher’s exact text. Statistical significance was set for all variables at p < 0.05.

## Results

In the study period between January 2012 and June 2019, 40 patients underwent either an MBG operation alone (MBG group) or an MBG procedure with IB (IB group). After applying the exclusion criteria, 23 patients were included in the study, with seven patients undergoing MBG with IB (IB group) and 16 undergoing an MBG procedure alone (MBG group). Ages ranged between 18 and 59 years, with an overall mean age of 36.1 years (SD = 13). The mean age of the IB group was 37.1 years (SD = 13.3) compared to 35.7 years (SD = 13.2) in the MBG alone. Overall, 60% of patients were female and 40% were male.

The mean MOxFQ overall raw index scores were significantly better in the IB group compared to the MBG group (10.9 vs. 33.6, p < 0.016) (Table 1). Standing and walking MOxFQ subscale (4 vs. 15.2, p < 0.012) and social interaction subscale (2 vs. 7.5 p < 0.023) both showed significantly better results for the IB group versus the MBG group. Moreover, the pain MOxFQ subscale (4.86 vs. 10.9, p < 0.042) demonstrated that the IB patients were in less pain in the long-term postoperative period. Furthermore, in the immediate postoperative period, 100% of the IB group patients reported pain had improved at their first clinic attendance compared to only 43.8% of the MBG group patients (p < 0.019). The median time from surgery to completion of the MOxFQ questionnaire was 1,142 days for the MBG group and 481 days for the IB group.

**Table 1:** MOxFQ scores for MBG with IB versus MBG alone – raw index scores. IB: internal brace; MBG: modified Broström-Gould; MOxFQ: Manchester-Oxford Foot Questionnaire

	MBG and IB	MBG alone	MBG and IB versus MBG (p < 0.05)
Overall	10.9	33.6	p < 0.016
Standing and walking subscale	4	15.2	p < 0.012
Social interaction subscale	2	7.5	p < 0.023
Pain subscale	4.86	10.9	p < 0.042

The IB group patients had significantly fewer weeks in a plaster cast postoperatively compared to the MBG group patients (4.14 vs. 6, p < 0.01) and were able to fully weight bear sooner (4.14 vs. 6, p < 0.01). The MBG group suffered a postoperative complication in seven patients compared to zero in the IB group (p < 0.057) (Table 2). There were three re-ruptures in the MBG group requiring further revision surgery compared to zero in the IB group (3 vs. 0, p < 0.53 (Table 2). The median time to assessment of complications postoperatively was 1,630 days for the IB group and 2,390 days for the MBG group.

**Table 2:** Postoperative complications MBG with IB versus MBG alone. IB: internal brace; MBG: modified Broström-Gould

	MBG and IB	MBG alone
Overall	0	7
Ligament re-rupture	0	3
Superficial wound infection	0	1
Deep wound infection	0	1
Complex regional pain syndrome	0	2
Deep vein thrombosis	0	0

There was no difference in average inpatient hospital length of stay postoperatively and no difference in the percentage of MBG versus IB patients discharged on the same day of the procedure (41.1% vs. 50%, p < 0.7).

## Discussion

Surgical technique development

Surgical intervention for CLAI which has failed conservative management can reduce pain, improve stability, and increase the likelihood of a successful return to pre-injury function [8]. The surgical technique has evolved from the Broström procedure to the MBG operation with or without an IB [4]. The Broström procedure was first hypothesized in 1966 and was the first surgical technique that used an anatomical approach to repair the ligaments [15]. Broström described repairing primarily the ATFL, directly and anatomically, as well as repairing the CFL if required [15]. Despite showing mainly positive results, this approach was altered by Gould in 1980 to include the repair of the extensor retinaculum and the LTCL as further reinforcement by overlapping the repaired ATFL (the primary lateral ankle stabilizer) [11]. Despite the MBG procedure being used by the majority of foot and ankle surgeons worldwide, more recent evidence has indicated that re-rupture rates may be higher than expected [16,17]. For patients with increased body mass index or those playing high-impact sporting activities, the stress placed through the repaired lateral ankle ligaments may exceed the strength of the repair from an MBG procedure [7]. It was hypothesized that augmenting the ATFL repair, the primary lateral ankle stabilizer, further may increase functional outcomes for patients with CLAI [18].

As the primary lateral ankle stabilizer, we must consider the strength of a native ATFL to that of a repaired ATFL. In biomechanical studies, the repaired ATFL in the MBG procedure is significantly less strong and less robust than the native ATFL [16,17]. Furthermore, evidence has demonstrated that MBG-repaired ligaments fail at the ligament-suture interface rather than at the ligament’s attachment to the talus or fibula [16]. Thus, the ATFL requires additional augmentation which spans the entire ligament increasing its strength and stability across the whole structure [16,17].

**Figure 2:**
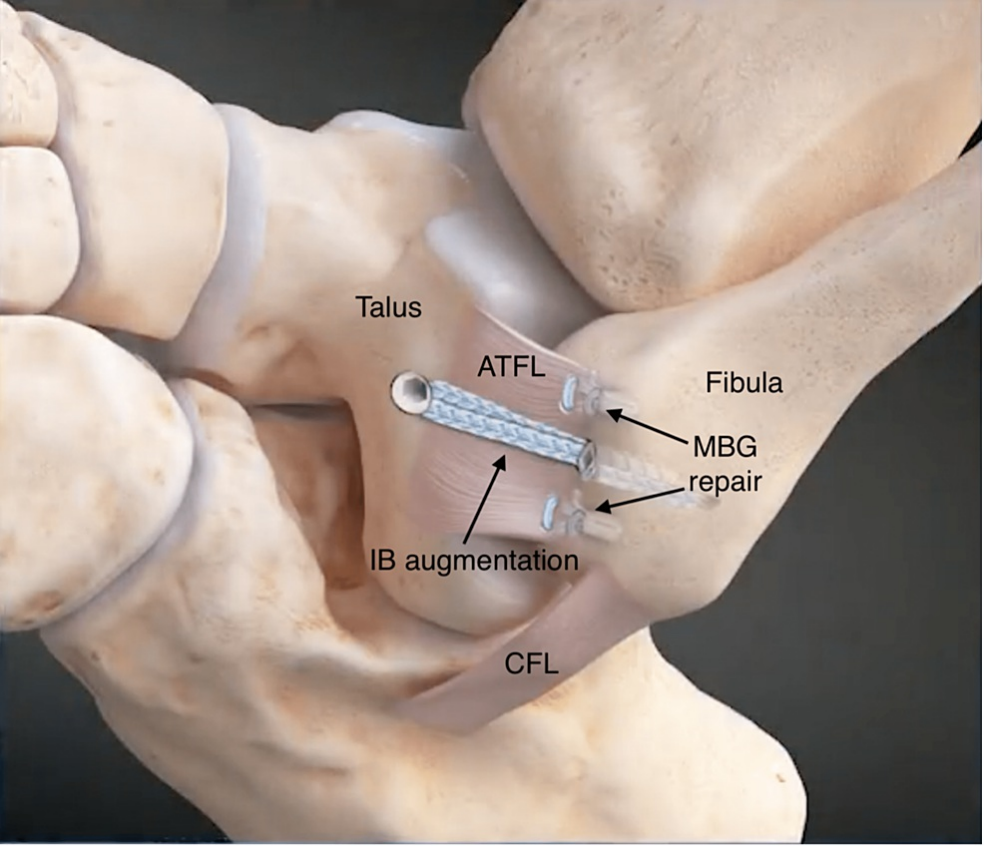
MBG repair with IB augmentation. Illustration based on the image from Arthrex case presentation video of MBG repair with IB by R J de Asla 2023 [19]. MBG repair performed using Knotless FiberTak® Anchors [18,19]. IB: internal brace; MBG: modified Broström-Gould

The technique of using IB augmentation in addition to MBG repair uses FiberTape which spans the entire repaired ATFL, with SwiveLocks to provide further stability (Figure 2). Biomechanical evidence has demonstrated the IB method to have more tensile strength and stiffness than MBG alone and no difference compared to the native ATFL [18].

Internal brace versus the modified Broström-Gould procedure

The results in this study suggest that the biomechanically stronger ATFL after MBG and IB augmentation demonstrates superior clinical functional outcomes (MOxFQ scores) than an MBG procedure alone (10.9 vs. 33.6, p < 0.016). The results demonstrated a significantly better MOxFQ overall score for IB patients compared to MBG alone. To our knowledge, although previous recent studies have demonstrated an improvement in MOxFQ scores with IB, this is the first paper to demonstrate a significant difference [7]. In contrast to larger studies in the recent literature, our case series had less strict inclusion criteria and included all patients undergoing either procedure under the care of all three foot and ankle consultants in our department [7]. We suggest that our recruitment criteria combined with the analysis of multiple surgeons’ outcomes are more representative of the outcomes of a routine foot and ankle orthopedic service in the UK. Therefore, despite the small number of patients, we feel these results add further confidence to existing evidence that IB augmentation provides superior clinical functional outcomes than MBG alone. Furthermore, this study also demonstrated that the IB technique was significantly better than the MBG for each subset (pain: 4.86 vs. 10.9, p < 0.042; social interaction: 2 vs. 7.5 p < 0.023; standing/walking: 4 vs. 15.2, p < 0.012). The pain MOxFQ subscores demonstrated patients were in significantly less pain in the long-term postoperative period. In addition, on analysis of pain in the immediate postoperative period, 100% of patients in the IB group reported pain to have improved in their first consultation compared to only 43.75% in the MBG group (p < 0.019). These results suggest pain was better both in the immediate and late postoperative period for IB patients. To our knowledge, this has not been previously reported in the literature.

A key finding in this study is that IB patients were out of plaster casts and able to fully weight bear significantly earlier than those in the MBG group (4.14 vs. 6 weeks, p < 0.01). To our knowledge, this has not been previously demonstrated in the published literature. We hypothesize that surgeons had more confidence in the stability of the IB group and thus removed the plaster and allowed full weight-bearing significantly earlier. Reducing time in plaster and allowing patients to fully weight bear sooner reduces the risk of several postoperative complications such as ankle stiffness and deep vein thrombosis.

Postoperative complications

The MBG group suffered a postoperative complication in seven patients compared to zero in the IB group (p < 0.057) (Table 2). Complications were considered for patients who developed an infection (deep or superficial), complex regional pain syndrome, deep vein thrombosis, or re-rupture of the lateral ankle ligaments. At the time of publishing, none of the IB group patients had a re-rupture compared to three of the MBG group patients, of whom all required surgical intervention (p < 0.53) (Table 2). However, it must be noted that the majority of the IB procedures were performed later in the study period and had a smaller time period postoperatively for complications to occur. This was demonstrated by the median time to assessment of complications being 1,630 days for the IB group compared to 2,390 days for the MBG group. An equal follow-up period with a much larger patient population is required before drawing any conclusions about complication rates and specifically re-rupture rates between the IB and MBG procedures.

The Manchester-Oxford Foot and Ankle Questionnaire as a functional outcome measure

The MOxFQ) (Figure 1 was used in this study to assess the mechanical and functional outcomes of the procedures for patients with CLAI. The MOxFQ scoring system which is split into three subdomains allows an analysis of more specific functional outcomes (pain, social interaction, and standing/walking) while also giving an index score to interpret the overall impact of the procedure [20]. The MOxFQ overall index score is both valid and reliable placing confidence in the overall results of this study [20].

Limitations

The main limitations of this study are its relatively small sample size and its retrospective nature resulting in a difference in follow-up duration between groups for the MOxFQ and complications outcomes. Despite having a small patient population, the data were collected from three consultant foot and ankle surgeons overseeing a number of trainee surgeons performing the procedures. Although we acknowledge that the small sample size limits the power and validity of any conclusions, using multiple surgeons increases the reliability of the results and reduces the risk of bias. We recognize that the IB group was followed up for a smaller period of time than the MBG for the MOxFQ and complications outcomes. We believe this is a consequence of surgeons having improved results with the IB and thus the majority of cases performed most recently used an IB. It is possible that the IB group has had fewer complications due to its smaller follow-up period. Nevertheless, as the body of evidence is growing for the use of IB in addition to MBG for CLAI, there is potential for recruitment in a large multi-center randomized control trial to make robust conclusions about functional outcomes and complication rates.

## Conclusions

This study suggests that patients with CLAI undergoing IB in addition to an MBG procedure have improved functional outcomes and possibly less pain in the immediate and long-term postoperative period. The use of an IB allowed patients to fully weight bear sooner and reduced time in a plaster cast postoperatively. The IB augmentation does not appear to increase the risk of complications; however, a longer follow-up period is required before making any definite conclusions. Although a large multi-center randomized control trial is required to demonstrate a higher level of confidence in IB, this paper contributes to the increasing body of evidence showing positive results for IB augmentation in addition to the MBG procedure for CLAI.
